# Consistently high baseline estimates for the proportion of human exposure to rural African malaria vector populations that occurred indoors

**DOI:** 10.1186/1475-2875-11-S1-P50

**Published:** 2012-10-15

**Authors:** Bernadette Huho, Olivier Briët, Aklilu Seyoum, Chadwick Sikaala, Nabie Bayoh, John Gimnig, Fredros Okumu, Diadier Diallo, Salim Abdulla, Thomas Smith, Gerry Killeen

**Affiliations:** 1 Biomedical and Environmental Thematic Group, PO Box 78373, Dares Salaam, United Republic of Tanzania; 2 University of Basel, Petersplatz 1, Basel, CH-4003, Switzerland; 3 Swiss Tropical and Public Health Institute, Basel, Switzerland; 4 Liverpool School of Tropical Medicine, Vector Group, Pembroke Place, Liverpool L3 5QA, UK; 5 National Malaria Control Centre, Chainama Hospital College Grounds, Off Great East road, P.O.Box 32509, Lusaka, Zambia; 6 Centre for Global Health Research, Kenya Medical Research Institute, P.O. Box 1578, Kisumu, Kenya; 7 Centers for Disease Control and Prevention, P.O. Box 1578, Kisumu, Kenya; 8 Division of Parasitic Diseases, Centers for Disease Control and Prevention, Atlanta, GA, 4770 Buford Highway, Mailstop F-42, Atlanta GA 30341, USA; 9 London School of Hygiene and Tropical Medicine, Disease Control and Vector Biology Unit, Keppel Street, WCIE 7HT, London UK; 10 Centre National de Recherche et de Formation Sur LePaludisme (CNRFP), Ouagadougou, Burkina Faso

## Background

Insecticide treated nets (ITNs) and indoor residual spraying (IRS) are highly effective options for controlling malaria transmission in Africa because the most important vectors, which are from the *Anopheles gambiae* complex and the *An. funestus* group, prefer biting humans who are indoors at night. It is feared that sustained large scale use of ITNs and IRS can cause these vectors to shift biting in place and time where ITNs and IRS are not effective.

## Materials and methods

Matched surveys of mosquito and human behavior from six rural sites in Burkina Faso, Tanzania, Zambia, and Kenya with ITN coverage ranging from 0.2% to 82.5% were used to calculate the proportion of human exposure to *Anopheles gambiae* sensu lato and *An. funestus* s.l. that occurs indoors (*π_i_*) as an indicator of the maximum level of personal protection that ITN use can provide. The proportion of mosquitoes caught indoors (*P_i_*) and between the first and last hours when most people are indoors (*P_fl_*) were also calculated as underlying indicators of vector preference for feeding indoors or at night, respectively.

## Results

The vast majority of human exposure to *Anopheles* bites occurred indoors (*π_i_* = 0.90-1.00). Neither *An. gambiae* s.l. nor *An. funestus* s.l. strongly preferred feeding indoors (*P_i_* = 0.46-0.63 and 0.22-0.72, respectively) but they overwhelmingly preferred feeding at times when most humans were indoors (*P_fl_* = 0.84-1.00 and 0.93-0.99, respectively).

## Conclusions

These quantitative summaries of behavioral interactions between humans and mosquitoes establish baseline values against which behaviour observed in residual vector populations exposed to high ITN or IRS coverage can be compared. Longitudinal monitoring of these quantities is vital to evaluate the effectiveness of ITNs and IRS and to evaluate the need for development of complementary measures targeting the outdoor-biting vectors.

